# Development and Validation of a Deep-Learning Model to Detect CRP Level from the Electrocardiogram

**DOI:** 10.3389/fphys.2022.864747

**Published:** 2022-05-30

**Authors:** Junrong Jiang, Hai Deng, Hongtao Liao, Xianhong Fang, Xianzhang Zhan, Shulin Wu, Yumei Xue

**Affiliations:** ^1^ Guangdong Cardiovascular Institute, Guangdong Provincial People’s Hospital, Guangdong Academy of Medical Sciences, Guangzhou, China; ^2^ Guangdong Provincial Key Laboratory of Clinical Pharmacology, Guangdong Provincial People’s Hospital, Guangdong Academy of Medical Sciences, Guangzhou, China

**Keywords:** deep learning, ECG, CRP, C-reactive protein, CNN, convolutional neural network, AI

## Abstract

**Background:** C-reactive protein (CRP), as a non-specific inflammatory marker, is a predictor of the occurrence and prognosis of various arrhythmias. It is still unknown whether electrocardiographic features are altered in patients with inflammation.

**Objectives:** To evaluate the performance of a deep learning model in detection of CRP levels from the ECG in patients with sinus rhythm.

**Methods:** The study population came from an epidemiological survey of heart disease in Guangzhou. 12,315 ECGs of 11,480 patients with sinus rhythm were included. CRP > 5mg/L was defined as high CRP level. A convolutional neural network was trained and validated to detect CRP levels from 12 leads ECGs. The performance of the model was evaluated by calculating the area under the curve (AUC), accuracy, sensitivity, specificity, and balanced F Score (F1 score).

**Results:** Overweight, smoking, hypertension and diabetes were more common in the High CRP group (*p* < 0.05). Although the ECG features were within the normal ranges in both groups, the high CRP group had faster heart rate, longer QTc interval and narrower QRS width. After training and validating the deep learning model, the AUC of the validation set was 0.86 (95% CI: 0.85–0.88) with sensitivity, specificity of 89.7 and 69.6%, while the AUC of the testing set was 0.85 (95% CI: 0.84–0.87) with sensitivity, specificity of 90.7 and 67.6%.

**Conclusion:** An AI-enabled ECG algorithm was developed to detect CRP levels in patients with sinus rhythm. This study proved the existence of inflammation-related changes in cardiac electrophysiological signals and provided a noninvasive approach to screen patients with inflammatory status by detecting CRP levels.

## 1 Introduction

In the past, inflammation has been proved to be an important pathophysiological mechanism for the occurrence and development of arrhythmias (Grune et al., 2021). The arrhythmogenic effects are mainly achieved through changes in electrophysiological properties, including ion channel disturbances, early and late afterdepolarization, as well as enhanced fibrosis and structural remodeling in cardiomyopathies (Vonderlin et al., 2019). C-reactive protein (CRP) and high sensitivity C reactive protein (hsCRP), as non-specific inflammatory markers, are associated with cardiovascular risk (Mozos et al., 2019; Tibaut et al., 2019). High CRP level is not only associated with the presence and future development of atrial fibrillation (Aviles et al., 2003), but also predictive of atrial fibrillation after surgery (Maesen et al., 2012). Moreover, CRP has been shown to be potentially associated with life-threatening ventricular arrhythmias and cardiac arrest (Gaaloul et al., 2016). Although inflammation has been shown to be a cause of arrhythmias, it is still unknown whether electrocardiographic features are altered in patients with inflammation.

With the development of deep learnings, artificial intelligence (AI) algorithm is gradually applied to cardiovascular disease (Attia et al., 2019a; Attia et al., 2019c; Kusunose et al., 2020). Electrocardiography (ECG) is an excellent substrate for deep learnings, because ECG data with finite complexity is obtained in consistent protocols and archived in usable digital formats. We hypothesized that the ECG characteristics of people with high CRP level may have changed and can be recognized by AI-enabled ECG algorithm. Therefore, we first try to develop and validate of a Deep-Learning Model to detect CRP Level from the electrocardiogram.

## 2 Methods

### 2.1 Data Sources and Study Population

**FIGURE 1:**
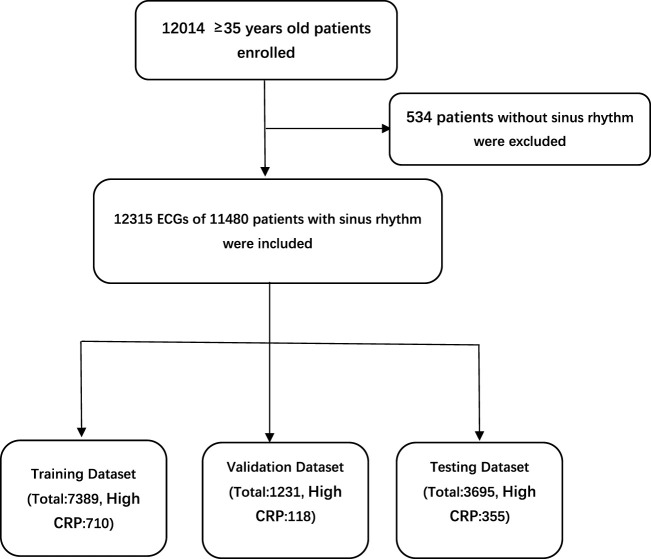
Patient flow diagram.

The study population came from an epidemiological survey of heart disease in Guangzhou, South of China. More than 12,000 adults aged 35 were enrolled in this survey. All underwent standard 10-s, 12-lead, 500-Hz ECG, and laboratory tests including CRP (detected by immunoturbidimetry), blood routine and electrolytes. 12,315 ECGs of 11,480 patients with sinus rhythm were included. The study protocol was approved by the ethics committee of Guangdong Provincial People’s Hospital and was conducted in accordance with the Declaration of Helsinki and Good Clinical Practice Guidelines. The requirement for informed consent from patients whose information was retrospectively collected was waived. According to the reference range of CRP in Guangdong Provincial People’s Hospital, CRP > 5 mg/L was defined as high CRP level. ECGs were divided into the high CRP group and the normal group. There were 1,183 ECGs in the high CRP group, and 11,132 ECGs in the normal group. ECGs were divided into the training set, validation set and testing set at proportions of 6:1:3, respectively (Figure 1).

### 2.2 Data Preprocessing

**FIGURE 2:**
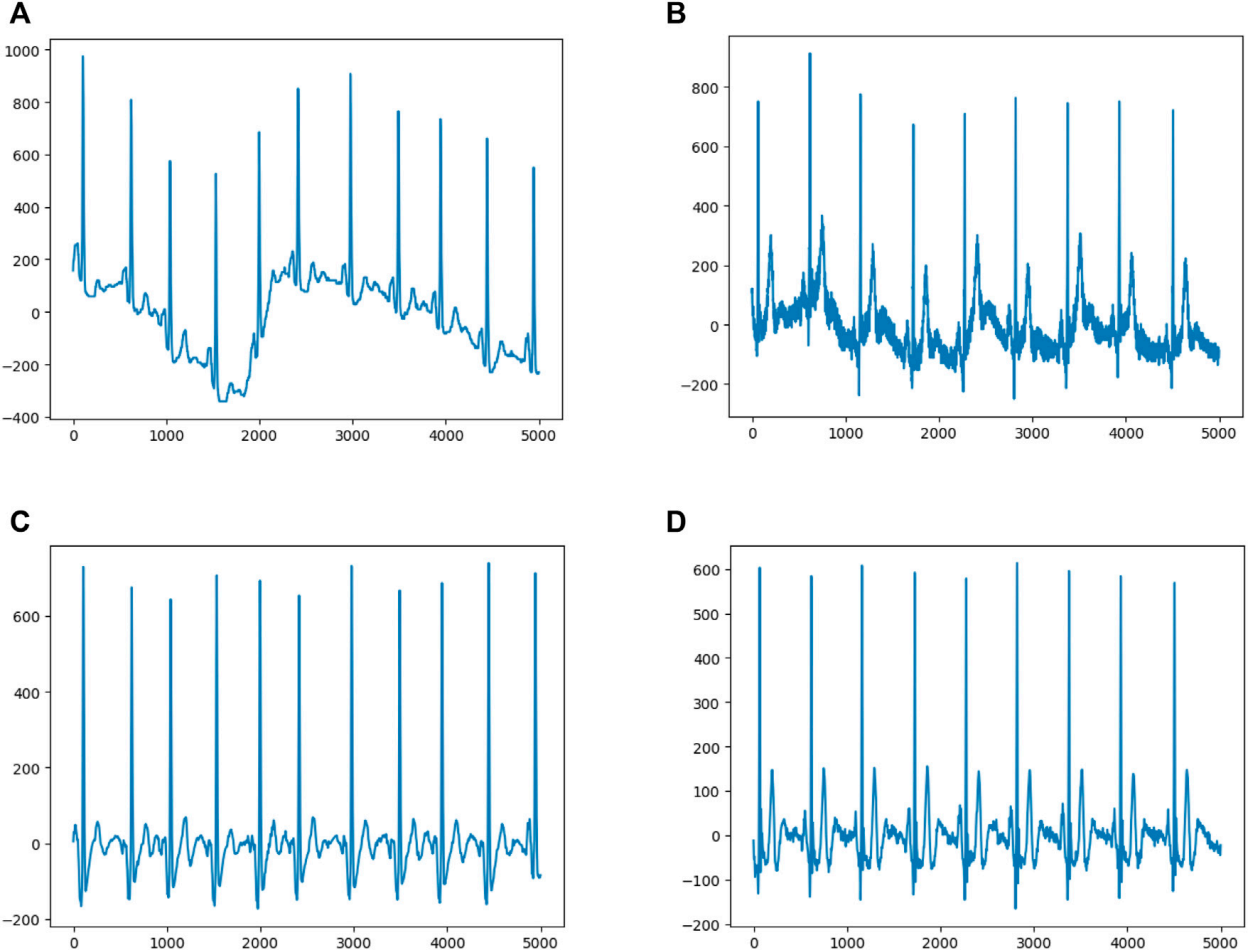
Data Preprocessing **(A)** and **(B)** show representative ECGs with baseline drift and noise, respectively **(C)** and **(D)** show representative ECGs after preprocessing.

The sample dimension was 12 × 5,000 (12 leads by 10-s duration sampled at 500 Hz). In order to remove the baseline drift (Figure 2A) and noise (Figure 2B), following our previous approach (Jiang et al., 2020), the raw data was first filtered by using a low-pass filter to get the baseline, then the baseline was flattened by zeroing the mean (Figure 2C), and denoising was achieved by filtering out the high frequency signal (Figure 2D). Since any linear function of the leads could be learned by the models and more artifacts were contained within the first and last 1-s periods, to optimize performance, only 8-s of eight independent leads (leads I, II, and V1–6) were selected. After preprocessing, each ECG was transformed to an 8 × 4,000 matrix.

### 2.3 Model Development

In order to produce more data and meanwhile to avoid affecting the evaluation of the testing set, the training and validation set were expanded 10 times by shifting the start point (Zhao et al., 2020). After data expanding, the training set contained 7,100 samples with high CRP level and 66,790 samples with normal CRP level while the validation set contained 1,180 samples with high CRP level and 11,130 samples with normal CRP level. To avoid training bias caused by the imbalance of samples, the same amounts of ECGs with different CRP levels were randomly selected as the input batch. ECGs in different dataset were not repeated.

Deep learning is an algorithm that can extract high-dimensional features from complex data and achieve classification. Multiple deep learning models were tested, and the simplest (the one with fewer parameters or layers) that resulted in the highest AUC was selected. The model that provided the optimal AUC was a two-dimensional convolutional neural network with a 12-layers, which was similar in concept and structure to a previously developed model detecting hyperkalaemia based on the 12-lead ECG (Galloway et al., 2019). The first 10 layers were convolutional to extract the subtle changes in ECGs and the last 2 layers formed a fully connected network to avoid overfitting. The output layer had two classes and was activated by “Softmax”. The model was built by the Keras Framework with TensorFlow backend and Python. Categorical cross entropy loss was used as the loss function, and Adam optimization method was applied.

### 2.4 Statistical Analysis

The continuous variables were compared by using the independent *t* test, and the 2-group categorical variables were compared by using the χ2 test and the log-rank test. Measures of performance included the area under curve (AUC), accuracy, precision, sensitivity, specificity, and balanced F Score (F1 score). Receiver operating characteristic curve (ROC) was obtained by using Python 3.5, Matplotlib 3.0.2 and ROC module. Statistical significance for differences was defined as a 2-sided *p* value of less than 0.05. Measures of performance were summarized using 2-sided 95% confidence intervals.

## 3 Results

### 3.1 Baseline Characteristics

The baseline clinical characteristics of the patients are shown in Table 1. There were 390 males and 715 females in the High CRP group with an average age of 60.5 years, while there were 3,604 males and 6,771 females in the normal group with an average age of 58.5 years. Patients in the High CRP group were older (*p* < 0.001), and no differences were shown in genders (*p* = 0.712). Overweight, smoking, hypertension and diabetes were more common in the High CRP group (*p* < 0.05). Consistent with CRP, the inflammatory markers such as leukocyte, platelet and neutrophil-to-lymphocyte ratio (NLR) were also significantly increased in the high CRP group (*p* < 0.001). Moreover, although the ECG features were within the normal ranges in both groups, the high CRP group had faster heart rate (*p* < 0.001), longer QTc interval (*p* < 0.001) and narrower QRS width (*p* = 0.03).

**TABLE 1:** Clinical Characteristics of Patients.

			ALL	High CRP	Normal	*p* Value
Basic information	Age (Mean) (Year)		58.5	60.5	58.3	<0.001
	Gender	Male	3,994	390 (9.8%)	3,604 (90.2%)	0.712
		Female	7486	715 (9.6%)	6771 (90.4%)	
	Overweight	yes	4151	546 (13.2%)	3,605 (86.8%)	<0.001
		no	7329	559 (7.6%)	6770 (92.4%)	
	Smoking	yes	2440	262 (10.7%)	2178 (89.3%)	0.038
		no	9040	843 (9.3%)	8197 (90.7%)	
	Alcohol	yes	2443	231 (9.5%)	2212 (90.5%)	0.748
		no	9037	874 (9.7%)	8163 (90.3%)	
Basic diseases	Hypertension	yes	3,367	417 (12.4%)	2950 (87.6%)	<0.001
		no	8113	688 (8.5%)	7425 (91.5%)	
	Diabetes	yes	1,042	139 (13.3%)	903 (86.7%)	<0.001
		no	10,438	966 (9.3%)	9472 (90.7%)	
	Stroke	yes	189	25 (13.2%)	164 (86.8%)	0.107
		no	11,291	1,080 (9.6%)	10,211 (90.4%)	
	Myocardial Infarction	yes	205	28 (13.7%)	177 (86.3%)	0.061
		no	11,275	1,077 (9.6%)	10,198 (90.4%)	
Laboratory Tests	CRP (mg/dl)		2.34	11.28	1.38	<0.001
	WBC(10^9/L)		6.94	8.21	6.80	<0.001
	PLT (10^9/L)		244.44	262.47	242.52	<0.001
	NLR		1.87	2.35	1.82	<0.001
ECG Features	heart rate (bpm)		70.50	74.07	70.12	<0.001
	PR interval (ms)		154.99	155.07	154.99	0.898
	QTc interval (ms)		432.47	439.36	431.73	<0.001
	QRS (ms)		86.64	85.88	86.73	0.030

CRP = C-reactive protein, WBC, white blood cell; PLT, platelet; NLR, neutrophil-to-lymphocyte ratio.

### 3.2 The Performance of the AI Algorithm

**FIGURE 3:**
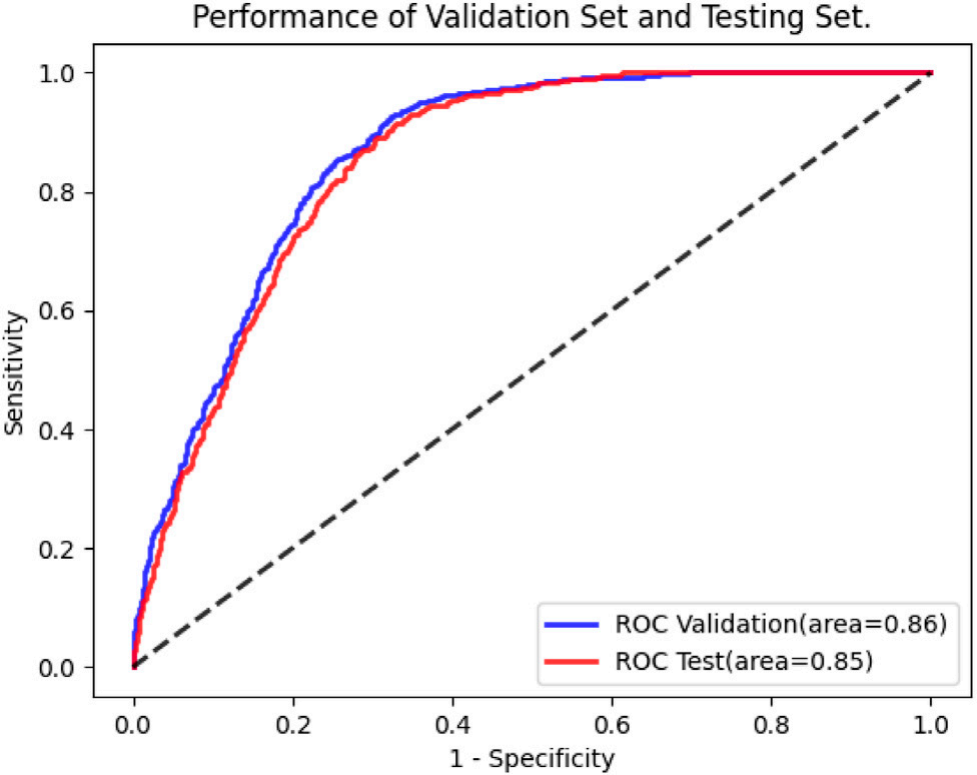
The performance of AI model.

Following training and validation, the ROC curves of detecting CRP were drawn (Figure 3) and the confusion matrix of the model was shown in Table 2. The AUC of the validation set was 0.86 (95% CI: 0.85–0.88). 821 of 1,180 ECGs with normal CRP level and 1,058 of 1,180 ECGs with high CRP level were detected by the AI model. The sensitivity, specificity, accuracy, precision, and F1 scores were 89.7, 69.6, 79.6, 74.7%, and 0.815, respectively (Table 2). The AUC of the testing set was 0.85 (95% CI: 0.84–0.87). 2,259 of 3,340 ECGs with normal CRP level and 322 of 355 ECGs with high CRP level were detected by the AI model. The sensitivity, specificity, accuracy, precision, and F1 scores were 90.7, 67.6, 69.9, 23.0%, and 0.366, respectively (Table 2). The results suggested that the AI model has a satisfactory ability to detect CRP level.

**TABLE 2:** The Confusion Matrix of AI Model.

		Predicted		Se (%)	Sp(%)	Acc(%)	Pre(%)	F1 Scores
		Normal	High CRP					
Validation Set	Normal	821	359	89.7	69.6	79.6	74.7	0.815
	High CRP	122	1,058					
Testing Set	Normal	2259	1,081	90.7	67.6	69.9	23.0	0.366
	High CRP	33	322					

Se = sensitivity; Sp = specificity; Acc = accuracy; Pre = precision.

## 4 Discussion

In this study, we found that AI-enabled ECG could effectively detect CRP level (AUC 0.85). The model performance was better than other common screening tests, such as B-type natriuretic peptide for heart failure (AUC 0.60–0.70) (Bhalla et al., 2005), mammography for breast cancer (AUC 0.78) (Pisano et al., 2005) and Papanicolaou smear for cervical cancer (AUC 0.70) (Chen et al., 2016). To our knowledge, this is the first deep-learning approach to evaluation of CRP levels from the ECGs.

Identification of patients with high CRP level by AI-enabled ECG indicated that although no obvious abnormality was observed, changes in cardiac electrophysiological signals caused by inflammation had already existed. Analysis of traditional ECG features suggested that patients with high CRP levels may have changes in QTc interval, heart rate, and QRS width compared with normal subjects. This also supported the existence of inflammation-related changes in cardiac electrophysiological signals. However, since these changes were still within the normal range, patients with high CRP levels could not be identified by these changes. The application of artificial intelligence to the ECG may enable noninvasive screening of patients with inflammatory status through the detection of CRP. This may be beneficial for patients with rheumatic immune diseases who need to evaluate the inflammatory status.

The AI-enabled ECG algorithm can not only be applied to diagnose and predict cardiovascular diseases (Ko et al., 2020; Raghunath et al., 2021), but also can be applied to other fields and realize the interpretation beyond manual. It is well known that some ECG features vary with age and gender differences, but none has particularly good discriminatory ability and there is no general agreement in routine ECG practice. In a study cohort of more than 275,000 patients, the AI-enabled ECG algorithm was able to predict patients’ sex (accuracy 90.4%, AUC 0.97) and age (mean error 6.9 ± 5.6 years) effectively (Attia et al., 2019b). Serum potassium levels are essential for normal cell function. Hyperkalemia significantly increases the risk of developing life-threatening arrhythmias, especially in those with cardiovascular or renal disease. The performance of AI-enabled ECG algorithm in detecting hyperkalemia was evaluated. After training using more than 1.5 million standard 12 lead ECGs from nearly 450,000 patients, the model was effective in detecting serum potassium levels in patients (AUC 0.853–0.883) (Galloway et al., 2019). This study suggested that the AI-enabled ECG algorithm was also valuable in detecting CRP levels. In the future, combined with deep learning, the application of ECG will be more promising.

In this study, aging, overweight, hypertension, diabetes and smoking seemed to be associated with high CRP levels. Previous studies have shown that aging and obesity are associated with inflammatory status (Franceschi et al., 2018; Kawai et al., 2021). Inflammation also plays a role in the pathogenesis of hypertension and diabetes (Lontchi-Yimagou et al., 2013; McMaster et al., 2015). In addition, the adverse consequences of smoking in various pathologies are mediated by its effects on the immune-inflammatory system (Rom et al., 2013). Although chronic inflammation was thought to be associated with alcohol-related diseases (Wang et al., 2010), the increase of inflammatory markers in people with alcohol dependence was not found in this study.

A total of over 12,000 ECGs were included to construct the deep learning model in this study. However, since the performance of deep learning models tends to increase as the amount of training data increases, the sample size of this study was still small. It is difficult to obtain such large amounts of labeled data in most situations. Previous studies have shown that deep learning models are also applicable in small datasets (Phillips et al., 2019; Makimoto et al., 2020). Zhao et al. developed an AI-based non ST segment elevation myocardial infarction (STEMI) auto diagnosis algorithm by using a dataset of 667 STEMI ECGs and 7571 control ECGs (Zhao et al., 2020). Makimoto et al. (11) successfully developed a convolutional neural networks model to recognize myocardial infarction by using the PTB ECG database consisting of 289 ECGs including 148 myocardial infarction cases (Makimoto et al., 2020).

## 5 Conclusion

In conclusion, an AI-enabled ECG algorithm was developed to detect CRP levels in patients with sinus rhythm. This study proved the existence of inflammation-related changes in cardiac electrophysiological signals and provided a noninvasive approach to screen patients with inflammatory status by detecting CRP levels. This model still requires further refinement and external validation.

## Data Availability

The raw data supporting the conclusions of this article will be made available by the authors, without undue reservation.
